# Supplementary material for: Effects of Ferroptosis on the Metabolome in Cardiac Cells: The Role of Glutaminolysis
Source: Antioxidants (Basel). 2022 Jan 29;11(2):278. doi: 10.3390/antiox11020278 (PMC8868370; doi:10.3390/antiox11020278)
Supplement: Supplementary file 1 [file antioxidants-11-00278-s001.zip › antioxidants-1527022-supplementary.pdf]

Supplementary Material

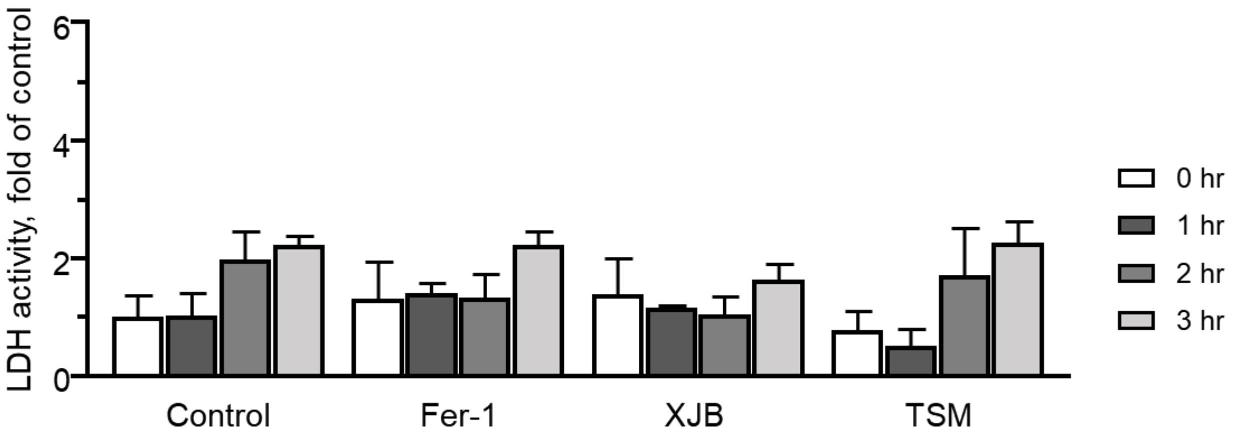

Figure S1. Time-dependence analysis of the effects of antioxidants (Fer-1, XJB, and TSM) on LDH activity in control (no RSL3) cells. LDH activity was measured in the culture medium containing H9c2 cells that were incubated with 1 µM Fer-1, 0.2 µM XJB, or 0.6 µM TSM for 1, 2, and 3 hr. Data were normalized to control for each time-point and presented as a fold increase. n=3 per group.

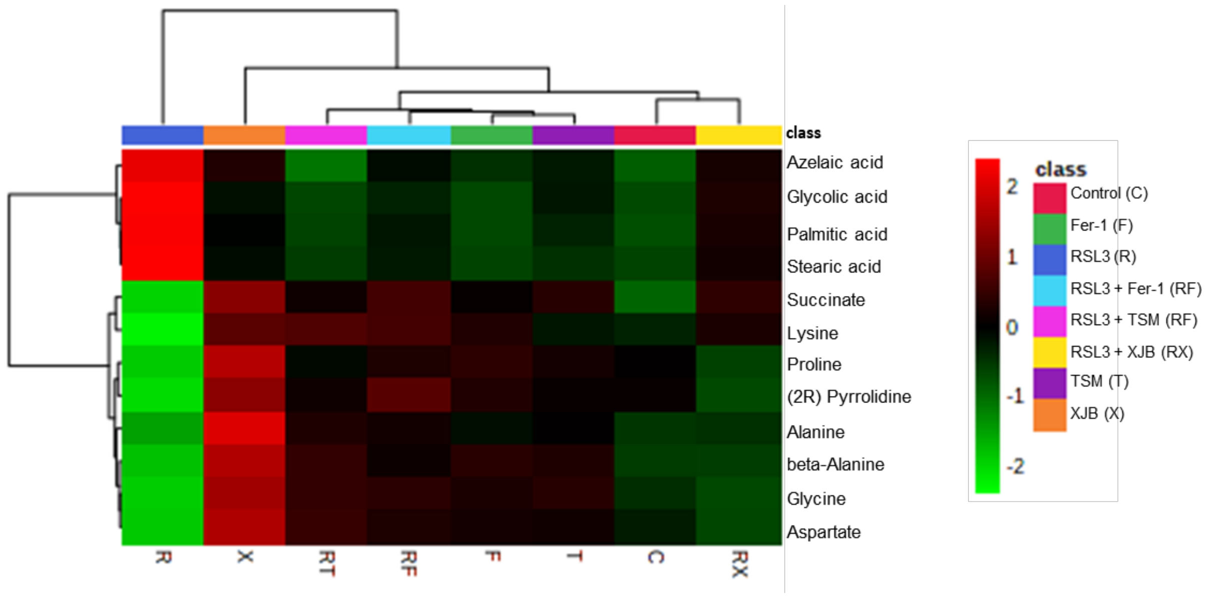

Figure S2. The metabolome of H9c2 cells exposed to RSL3 in the presence and absence of Fer-1, XJB or TSM. Heatmap displays average metabolites concentration (mM) for each group as compared to control and RSL3 groups after Bonferroni correction. The conditions (in the abscise axis) and the bins/metabolites (in the ordinate axis) have been sorted according to cluster analysis. The fold change is color-coded according to the bar legend. C, control; R, RSL3; F, Fer-1; X, XJB; T, TSM; RF, RSL3+Fer-1; RX, RSL3+XJB; RT, RSL3+TSM. Green represents lower concentration; red represents higher concentrations. n=4-6 per group.

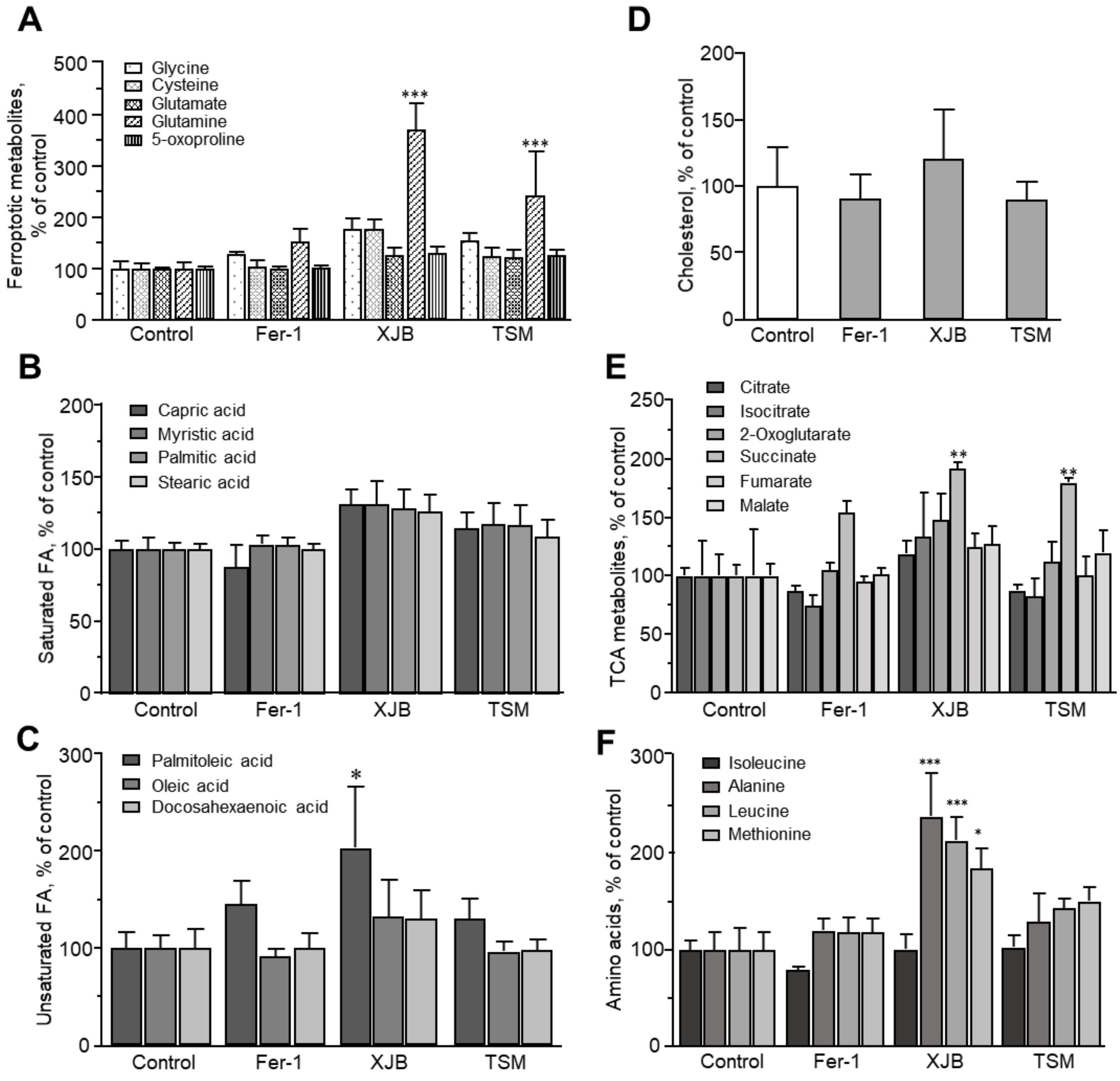

Figure S3. The effects of antioxidants (Fer-1, XJB and TSM) on GSH precursor metabolites, saturated and unsaturated FA, cholesterol, TCA metabolites and amino acids in control (no RSL3) cells. Glycine, cysteine, glutamate, and glutamine represent GSH precursors, while 5-oxoproline is a marker of GSH degradation within the cell (***A***). Saturated FA include capric, myristic, palmitic, and stearic acid (***B***). Unsaturated FA include palmitoleic, oleic, and docosahexaenoic acid (***C***). Cholesterol levels in control and antioxidant-treated cells (***D***). The TCA metabolites (***E***) identified were citrate, isocitrate, 2-oxoglutarate, succinate, fumarate, and malate. The amino acids identified are isoleucine, alanine, leucine, and methionine (***F***). Data are presented as percentage of control. $^{*}p<0.05$, $^{**}p<0.01$ and $^{***}p<0.001$ *vs* control. n=4-6 per group.

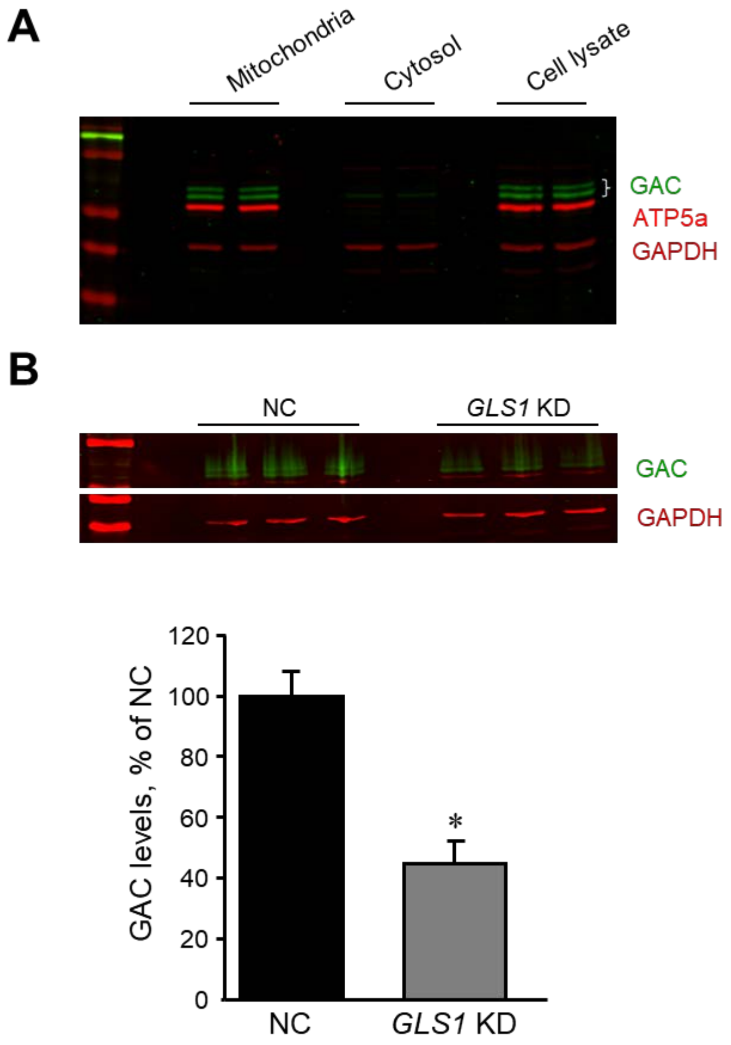

Figure S4. Mitochondrial *GLS1* silencing in H9c2 cells. Representative immunoblots (*top*) that represent localization of GAC in mitochondria isolated from H9c2 cells (***A***). Immunoblot representing GAC in non-coding (NC) and *GLS1* KD cardiomyocytes (***B***). ATP5A and GAPDH expression were used to validate the mitochondrial expression of GAC. Expression of GAC was normalized to GAPDH. Quantitative data of GAC from NC and *GLS1* KD are presented as percentage of NC. $^{*}p<0.05$ *vs* NC. n=3 per group.

Table S1. Characterization of identified metabolites

ID		Fragment Ions	Retention time	HMDB	PubChem	KEGG	Formula	Exact mass	Super class	Main class	Sub class
1.	Alanine	158, 232, 260	10.754	HMDB0000161	5950	C00041	C3H7NO2	89.0477	Organic acids	Amino acids and peptides	Amino acids
2.	Glycine	218, 246	11.071	HMDB0000123	750	C00037	C2H5NO2	75.032	Organic acids	Amino acids and peptides	Amino acids
3.	beta-Alanine	144, 218, 260	12.236	HMDB0000056	239	C00099	C3H7NO2	89.0477	Organic acids	Amino acids and peptides	Amino acids
4.	Valine	186, 260, 288	12.471	HMDB0000883	6287	C00183	C5H11NO2	117.079	Organic acids	Amino acids and peptides	Amino acids
5.	Leucine	200, 274, 302	13.04	HMDB0000687	6106	C00123	C6H13NO2	131.0946	Organic acids	Amino acids and peptides	Amino acids
6.	Isoleucine	200, 274, 302	13.48	HMDB0000172	6306	C00407	C6H13NO2	131.0946	Organic acids	Amino acids and peptides	Amino acids
7.	L-Isoleucine	86, 170, 188	8.737	HMDB0000172	6306	C00407	C6H13NO2	131	Organic acids	Amino acids and peptides	Amino acids
8.	Proline	184, 258, 286	13.991	HMDB0000162	145742	C00148	C5H9NO2	115.0633	Organic acids	Amino acids and peptides	Amino acids
9.	Sarcosine	158, 232, 260	14.873	HMDB0000271	1088	C00213	C3H7NO2	89.0477	Organic acids	Amino acids and peptides	Amino acids
10.	Methionine	218, 292, 320	16.551	HMDB0000696	6137	C00073	C5H11NO2S	149.0511	Organic acids	Amino acids and peptides	Amino acids
11.	N-Acetylaspartic acid	73, 225, 346	16.601	HMDB0000812	65065	C01042	C6H9NO5	175.0481	Organic acids	Amino acids and peptides	Amino acids
12.	N-Acetylglutamic acid	186, 216, 318	16.8	HMDB0001138	185	C00624	C7H11NO5	189.0637	Organic acids	Amino acids and peptides	Amino acids
13.	Serine	288, 362, 390	16.823	HMDB0000187	5951	C00065	C3H7NO3	105.0426	Organic acids	Amino acids and peptides	Amino acids
14.	Threonine	303, 376, 404	17.212	HMDB0000167	6288	C00188	C4H9NO3	119.0582	Organic acids	Amino acids and peptides	Amino acids
15.	DL-Phenylalanine	120, 130, 146	14.32	HMDB0250791	994	C02265	NA	165.079	Organic acids	Amino acids and peptides	Amino acids
16.	Phenylalanine	234, 302, 336	18.02	HMDB0000159	6140	C00079	C9H11NO2	165.079	Organic acids	Amino acids and peptides	Amino acids
17.	Aspartic acid	302, 316, 418	18.933	HMDB0000191	5960	C00049	C4H7NO4	133.0375	Organic acids	Amino acids and peptides	Amino acids
18.	Cysteine	302, 378, 406	19.315	HMDB0000574	5862	C00097	C3H7NO2S	121.0198	Organic acids	Amino acids and peptides	Amino acids
19.	Glutamic acid	272, 330, 432	20.026	HMDB0000148	33032	C00025	C5H9NO4	147.0532	Organic acids	Amino acids and peptides	Amino acids
20.	Asparagine	302, 400, 417	20.361	HMDB0000168	6267	C00152	C4H8N2O3	132.0535	Organic acids	Amino acids and peptides	Amino acids
21.	Lysine	198, 272, 300	21.144	HMDB0000182	5962	C00047	C6H14N2O2	146.1055	Organic acids	Amino acids and peptides	Amino acids
22.	Glutamine	198, 271, 431	21.596	HMDB0000641	5961	C00064	C5H10N2O3	146.0691	Organic acids	Amino acids and peptides	Amino acids
23.	Tyrosine	302, 364, 466	23.7	HMDB0000158	6057	C00082	C9H11NO3	181.0739	Organic acids	Amino acids and peptides	Amino acids
24.	Urea	73, 147, 231	12.371	HMDB0000294	2447	C00086	CH4N2O	60.0324	Organic acids	Organic carbonic acids	Ureas
25.	Glycolic acid	189, 247	10.282	HMDB0000115	757	C03547	C2H4O3	76.016	Fatty Acyls	Fatty acids	Hydroxy FA
26.	Capric acid	75, 229	12.83	HMDB0000511	2969	C01571	C10H20O2	172.1463	Fatty Acyls	Fatty acids	Saturated FA
27.	Azelaic acid	129, 359, 401	17.57	HMDB00784	2266	C08261	C9H16O4	188.1049	Fatty Acyls	Fatty Acids	Saturated FA
28.	Myristic acid	75, 129, 285	17.892	HMDB0000806	11005	C06424	C14H28O2	228.2089	Fatty Acyls	Fatty acids	Saturated FA
29.	Palmitoleic acid	75, 311, 353	20.051	HMDB0003229	5312427	C08362	C16H30O2	254.2246	Fatty Acyls	Fatty acids	Saturated FA
30.	Palmitic acid	75, 117, 129, 313	20.12	HMDB0000220	985	C00249	C16H32O2	256.2402	Fatty Acyls	Fatty acids	Saturated FA
31.	Oleic acid	75, 129, 339	21.946	HMDB0000207	445639	C00712	C18H34O2	282.2559	Fatty Acyls	Fatty acids	Unsaturated FA
32.	Stearic acid	75, 341	22.176	HMDB0000827	5281	C01530	C18H36O2	284.2715	Fatty Acyls	Fatty acids	Saturated FA
33.	Docosahexaenoic acid	75, 209, 385, 427	25.482	HMDB0002183	445580	C06429	C22H32O2	328.2402	Fatty Acyls	Fatty acids	Unsaturated FA
34.	Pantothenic acid	75, 87, 504	24.138	HMDB0000210	6613	C00864	C9H17NO5	219.11	Organic Oxygen	Organooxygen	Secondary alcohols
35.	Erythronic acid	261, 344, 476	24.56	HMDB0000613	2781043	NA	C4H8O5	136.0372	Carbohydrates	Monosaccharides	Sugar acids
36.	Glycerol	245, 289, 377	15.43	HMDB00131	753	C00116	C3H8O3	92.0473	Organic Oxygen	Organooxygen	Sugar alcohol
37.	Hypoxanthine	73, 193, 307	19.554	HMDB0000157	790	C00262	C5H4N4O	136.0385	Nucleic acids	Purines	Hypoxanthines
38.	Nicotinamide	75, 136, 180	13.209	HMDB0001406	936	C00153	C6H6N2O	122.048	Organoheterocyclic compounds	Pyridinecarboxylic acids	Nicotinamides
39.	Uracil	75, 179, 195	11.632	HMDB0000300	1174	C00106	C4H4N2O2	112.0273	Nucleic acids	Pyrimidines	Pyrimidines
40.	5-oxoprolinate	84, 158, 186	13.578	HMDB0000805	499	C02237	C5H7NO3	129.0426	Organoheterocyclic compounds	Pyrroline carboxylic acids	Pyrroline carboxylic acids
41.	5-oxoproline	198, 272, 300	16.349	HMDB0000267	7405	C01879	C5H7NO3	129.0426	Organoheterocyclic compounds	Pyrroline carboxylic acids	Pyrroline carboxylic acids
42.	(2R)-Pyrrolidine	184, 214, 330	17.487	NA	91742919	NA	C18H37NO4Si2	387.7	Organoheterocyclic compounds	Pyrroline carboxylic acids	Pyrroline carboxylic acids
43.	Lactic acid	189, 233, 261	10.048	HMDB0000190	61503	C00256	C3H6O3	90.0317	Organic acids	Short-chain acids	Short-chain acids
44.	Cholesterol	75, 367, 443	35.95	HMDB0000067	5997	C00187	C27H46O	386.3549	Sterol Lipids	Sterols	Cholesterols
45.	Taurine	88, 158, 296	15.844	HMDB0000251	1123	C00245	C2H7NO3S	125.0147	Organic acids	Sulfonic acids	Sulfonic acids
46.	Phosphoric acid	124, 383, 384	16.013	HMDB0001429	1004	C00009	H3PO4	97.9769	Non-metal compounds	Non-metal oxanionic compounds	Non-metal phosphates
47.	Succinic acid	73,147, 289, 331	14.028	HMDB0000254	1110	C00042	C4H6O4	118.0266	Organic acids	TCA acids	TCA acids
48.	Fumarate	147, 287, 329	17.02	HMDB00134	444972	C00122	C4H4O4	116.011	Organic acids	TCA acids	TCA acids
49.	Malate	115, 287, 419	18.233	HMDB0000744	525	C03668	C4H6O5	134.0215	Organic acids	TCA acids	TCA acids
50.	2-Oxoglutarate	157, 215, 317	19.22	HMDB0000208	51	C00026	C5H6O5	146.0215	Organic acids	TCA acids	TCA acids
51.	Citrate	431, 459, 591	23.494	HMDB0000094	311	C00158	C6H8O7	192.027	Organic acids	TCA acids	TCA acids
52.	Isocitrate	73, 147, 202, 345	24.435	HMDB0000193	1198	C00311	C6H8O7	192.027	Organic acids	TCA acids	TCA acids

Table S2. Statistics and fold of change of identified metabolites

				Fold of Change						
**Metabolite**	***p*-value***	***f*-value**	**FDR**	**R vs C**	**R vs F**	**R vs X**	**R vs T**	**R vs RF**	**R vs RX**	**R vs RT**
1. Alanine	0.000465	5.0466	0.002418	0.45137	0.37607	0.18929	0.35014	0.32965	0.43809	0.31546
2. Glycine	0.02179	2.7411	0.049265	0.19123	0.13228	0.091055	0.12677	0.12213	0.21731	0.099866
3. beta-Alanine	0.000287	5.3627	0.001866	0.1929	0.15826	0.099807	0.16058	0.15327	0.23905	0.13984
4. Valine	0.000257	5.4369	0.001866	0.24701	0.1578	0.10604	0.16462	0.17595	0.25106	0.1528
5. Leucine	0.007599	3.3409	0.024698	0.35956	0.28363	0.16565	0.29166	0.25887	0.41709	0.2798
6. Isoleucine	0.60092	0.78927	0.62496	0.98795	1.3862	1.1175	0.9781	0.88436	1.4176	1.5679
7. L-Isoleucine	0.037546	2.4367	0.072311	0.56393	0.49211	0.38737	0.49741	0.44157	0.52644	0.44593
8. Proline	0.004702	3.6207	0.017465	0.28874	0.15206	0.074829	0.11893	0.14032	0.22088	0.1046
9. Sarcosine	0.000354	5.2243	0.002046	0.3995	0.31348	0.22418	0.30365	0.29913	0.44146	0.29343
10. Methionine	0.01659	2.8947	0.041622	0.7699	0.64735	0.36015	0.64545	0.5003	0.72852	0.62107
11. N-Acetylaspartic acid	0.019701	2.7978	0.046566	0.82832	0.6981	0.38823	0.70483	0.55032	0.7638	0.6565
12. N-Acetylglutamic acid	0.52812	0.88499	0.57147	1.1664	1.471	1.1613	1.1406	0.99626	1.5696	1.605
13. Serine	0.002185	4.0779	0.009468	0.000472	0.000355	0.000296	0.000445	0.000315	0.000364	0.000306
14. Threonine	0.025802	2.6462	0.052951	0.69375	0.58752	0.37516	0.55415	0.45267	0.70688	0.53603
15. DL-Phenylalanine	0.44528	1.003	0.50336	1.4925	1.5375	1.127	1.3296	1.0458	1.2883	1.2784
16. Phenylalanine	0.21984	1.4405	0.32662	0.64705	0.69121	0.59704	0.59199	0.58943	0.76147	0.66882
17. Aspartic acid	0.026475	2.6318	0.052951	0.6764	0.55633	0.34423	0.58467	0.46216	0.68584	0.52749
18. Cysteine	3.47E-05	6.8366	0.000433	0.000238	0.000197	0.000128	0.000219	0.000212	0.000355	0.000252
19. Glutamic acid	0.045525	2.3293	0.084546	0.000581	0.001381	0.001788	0.000835	0.000456	0.001617	0.00051
20. Asparagine	0.010846	3.1364	0.031333	0.60375	0.74758	0.51682	0.48858	0.50718	0.73506	0.51826
21. Lysine	0.002986	3.8899	0.011946	0.38805	0.34158	0.2292	0.29799	0.26614	0.39116	0.25894
22. Glutamine	0.11779	1.7984	0.19758	0.81884	0.68182	0.43508	0.7074	0.57742	0.77872	0.62213
23. Tyrosine	0.055225	2.2218	0.099025	0.97292	0.83104	0.50012	0.85398	0.65368	0.90055	0.82471
24. Urea	0.32319	1.2088	0.40014	1.1075	1.0997	0.64372	1.0435	0.82103	1.2533	1.2083
25. Glycolic acid	0.005742	3.5037	0.019905	2.3734	2.7078	1.8082	2.0671	1.9256	1.6432	2.2905
26. Capric acid	0.31347	1.2276	0.40014	1.2454	1.2416	0.95396	1.2768	0.805	1.1376	1.7431
27. Azelaic acid	1.06E-05	7.734	0.000276	2.6263	2.6005	2.0449	2.0847	2.211	1.7472	2.5882
28. Myristic acid	0.009365	3.2205	0.028647	1.8381	1.7802	1.4001	1.5659	1.5228	1.419	1.8331
29. Palmitoleic acid	0.42477	1.0344	0.49084	1.6845	1.8374	1.5878	1.7512	1.3004	1.4191	1.831
30. Palmitic acid	4.16E-05	6.7047	0.000433	2.2618	2.1988	1.7549	1.9399	1.8504	1.6213	2.1604
31. Oleic acid	0.35335	1.153	0.42731	1.5926	1.0924	0.78291	1.2217	1.2425	1.7377	1.5305
32. Stearic acid	2.71E-06	8.8408	0.000141	2.5305	2.525	2.001	2.3283	2.1007	1.7921	2.4322
33. Docosahexaenoic acid	0.000163	5.7449	0.001408	2.2962	1.9396	1.5314	1.8022	1.7361	1.5706	2.5398
34. Pantothenic acid	0.28645	1.2827	0.38194	2.2998	3.1513	1.8185	3.3046	1.1078	2.3149	2.5826
35. Erythronic acid	0.16206	1.6172	0.24785	1.6191	1.6229	1.3566	1.3703	1.4073	1.2689	1.4847
36. Glycerol	0.016809	2.8873	0.041622	1.957	2.0572	1.8127	1.4226	1.5894	1.5685	1.7877
37. Hypoxanthine	0.22754	1.4203	0.32867	1.1823	1.2588	0.67865	0.77958	0.79017	0.87667	0.77361
38. Nicotinamide	0.13347	1.7278	0.21033	0.32507	0.28327	0.21812	0.23745	0.23077	0.36964	0.21747
39. Uracil	0.31647	1.2217	0.40014	0.85145	0.67819	0.5304	0.75509	0.66592	0.82839	0.74868
40. 5-oxoprolinate	0.39199	1.0868	0.46326	0.94553	1.3068	1.1293	1.5626	1.2069	1.2157	1.6754
41. 5-oxoproline	0.00054	4.9504	0.002552	0.37115	0.34784	0.27225	0.3703	0.30489	0.47757	0.36355
42. (2R)-Pyrrolidine	0.5385	0.87098	0.57147	0.7844	1.2809	1.2132	0.6724	0.784	1.2053	1.3607
43. Lactic acid	0.6719	0.69981	0.68507	0.95744	0.94621	0.73264	0.92467	0.79855	0.98463	0.84251
44. Cholesterol	0.023495	2.6987	0.050907	1.6771	1.7759	1.3832	1.4606	1.4441	1.6606	1.732
45. Taurine	0.89159	0.40708	0.89159	1.575	1.7294	1.3052	1.7509	1.3196	1.6924	1.5237
46. Phosphoric acid	0.013254	3.022	0.036274	0.003135	2.6869	0.01528	0.00454	0.032471	1.8459	2.4323
47. Succinic acid	0.075114	2.0505	0.1302	1.7281	1.6461	1.1658	1.5381	1.4972	1.5413	1.7869
48. Fumarate	0.27536	1.3066	0.38194	1.404	1.3857	1.0112	1.7191	1.0962	1.2708	1.4542
49. Malate	0.27925	1.2981	0.38194	1.5184	1.595	1.2156	1.507	1.5141	1.3863	1.5939
50. 2-Oxoglutarate	0.13348	1.7278	0.21033	2.3038	3.0796	1.7249	2.788	1.1437	2.3368	2.6068
51. Citrate	0.50409	0.918	0.55772	0.87385	0.86159	0.68413	0.72851	0.73753	0.93917	0.86398
52. Isocitrate	1.83E-05	7.3164	0.000317	0.44452	0.28792	0.20215	0.26053	0.2404	0.25537	0.28154

Table S3. Significant differences in metabolite concentrations between groups

Metabolite	Difference between groups	*p*-value*	*f*-value	FDR
1. Stearic acid	**R** vs. C, F, RF RT, RX, T, X	2.71E-06	8.8408	0.000141
2. Glycolic acid	**R** vs. C, F, RF RT, RX, T, X	1.06E-05	7.734	0.000276
3. Succinic Acid	**C** vs. RF, RX, T, X<br>**R** vs. F, RF, RT, RX, T, X<br>**X** vs. F, RT	1.83E-05	7.3164	0.000317
4. Proline	**C** vs. R X<br>**R** vs. F, RF, RT, RX, T, X<br>**X** vs F, RF, RT, RX, T	3.47E-05	6.8366	0.000433
5. Palmitic Acid	**R** vs. C, F, RF, RT, RX, T, X	4.16E-05	6.7047	0.000433
6. Azelaic acid	**R** vs. C, F, RF, RT, RX, T, X<br>**RX** vs. RT<br>**X** vs. RT	0.000163	5.7449	0.001408
7. beta-Alanine	**C** vs. R, X<br>**R** vs. F, RF, RT, RX, T, X<br>**X** vs. F, RF, RX, T	0.000257	5.4369	0.001866
8. Aspartic acid	**C** vs R, X<br>**R** vs. F, RF, RT, RX, T, X<br>**X** vs F, RF, RX, T	0.000287	5.3627	0.001866
9. Glycine	**C** vs R, X<br>**R** vs F, RF, RT, RX, T, X<br>**X** vs. RX	0.000354	5.2243	0.002046
10. Alanine	**C** vs X<br>**R** vs. F, RF, RT, T, X<br>**X** vs F, RF , RT, RX, T	0.000465	5.0466	0.002418
11. (2R)-Pyrrolidine	**R** vs C, F, RF, RT, RX, T, X<br>**RX** vs RF, X	0.00054	4.9504	0.002552
12. Lysine	**R** vs C, F, RF, RT, RX, T, X	0.002185	4.0779	0.009468
